# Efficient Tree Searches with Available Algorithms

**Published:** 2007-11-12

**Authors:** Gonzalo Giribet

**Affiliations:** Department of Organismic and Evolutionary Biology and Museum of Comparative Zoology, Harvard University, 26 Oxford Street, Cambridge, MA 02138, U.S.A

## Abstract

Phylogenetic methods based on optimality criteria are highly desirable for their logic properties, but time-consuming when compared to other methods of tree construction. Traditionally, researchers have been limited to exploring tree space by using multiple replicates of Wagner addition followed by typical hill climbing algorithms such as SPR or/and TBR branch swapping but these methods have been shown to be insuficient for “large” data sets (or even for small data sets with a complex tree space). Here, I review different algorithms and search strategies used for phylogenetic analysis with the aim of clarifying certain aspects of this important part of the phylogenetic inference exercise. The techniques discussed here apply to both major families of methods based on optimality criteria—parsimony and maximum likelihood—and allow the thorough analysis of complex data sets with hundreds to thousands of terminal taxa. A new technique, called *pre-processed searches* is proposed for reusing phylogenetic results obtained in previous analyses, to increase the applicability of the previously proposed *jumpstarting phylogenetics* method. This article is aimed to serve as an educational and algorithmic reference to biologists interested in phylogenetic analysis.

## Rationale

In phylogenetic analysis, numerical methods are preferred over other methods because of their efficiency and repeatability. Within numerical methods, those based on optimality criteria are to be preferred because they allow for hypothesis testing and tree comparisons based on objective measures. However, methods based on optimality criteria are more time consuming than most other numerical methods (e.g. UPGMA, neighbor-joining). The reason for this is simple, in order to choose an optimal solution, multiple trees need to be compared. The two main optimality criteria are parsimony and maximum likelihood^1^. While their limits on efficient searches differ due to the computation requirements by each method (e.g. Sanderson and Kim, 2000; Goloboff, 2003), the issues discussed in this article apply, at least in principle, to both methodologies.

Finding the optimal tree(s) for a given optimality criterion—the so-called “tree search”—is a NP-complete problem (Garey et al. 1977; Garey and Johnson, 1977; Chor and Tuller, 2005); a problem that is unlikely to have a solution in polynomial time. Tree searches are difficult due to the exponential growth of possible trees when increasing the number of terminals (OTUs) (Felsenstein, 1978). If a method were to compare all the possible trees using an explicit enumeration technique, an optimality value (tree length for parsimony or −lnL score for maximum likelihood) would be assigned to each tree and those that optimize the selected criterion would be chosen. However, explicit enumeration is not a very efficient method and there are many algorithmic speedups that will find the optimal solution without the burden of evaluating all possible trees. An alternative solution to explicit enumeration is the use of shortcuts that guarantee finding all optimal trees. The most common shortcut is the *branch and bound* algorithm (Hendy and Penny, 1982). Although for teaching purposes explicit enumeration and branch and bound do the trick, for most biologically interesting datasets these algorithms cannot be usefully applied. Instead, most investigators use different types of heuristics to attempt achieving an optimal—if not “the” optimal—solution by avoiding the intractability of exact methods. Heuristic methods cannot guarantee finding the optimal solution, unlike exact methods, although convergence measures can be used (see below) as indicators of the quality of the result.

**Figure 1.**
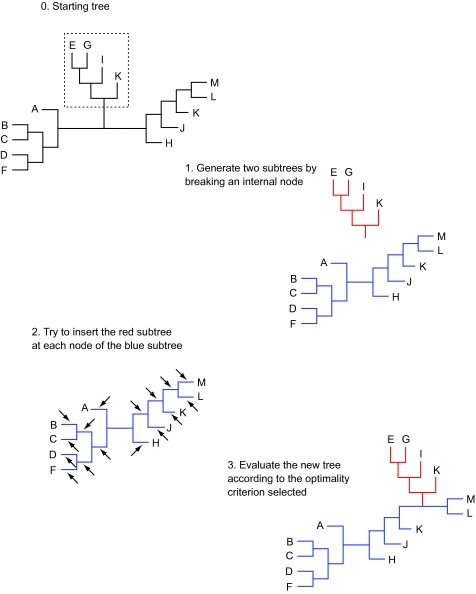
SPR branch swapping. An initial tree (0) gets broken into two subtrees (1). The red subtree is then inserted in each possible branch of the blue subtree (arrows in step 2) and the resulting tree is evaluated (3).

**Figure 2.**
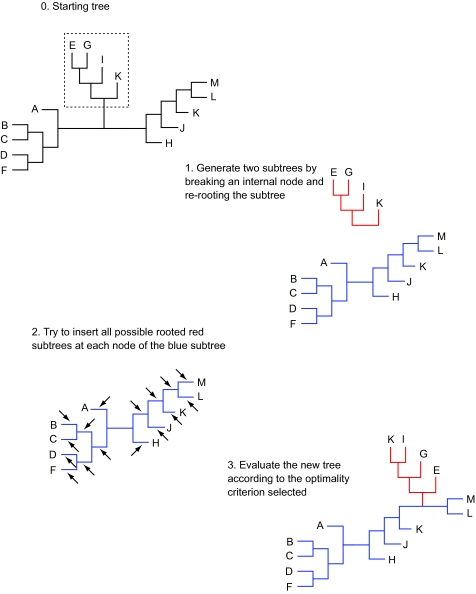
TBR branch swapping. An initial tree (0) gets broken into two subtrees (1). The red subtree is re-rooted on each possible internal branch and inserted in each possible branch of the blue subtree (arrows in step 2) and the resulting tree is evaluated (3).

Historically, the first heuristic method of tree construction is the algorithm proposed by Wagner (1961) and implemented by Farris (1970). Such trees were originally calculated by hand and used as *final* results to interpret a phylogeny. But it became evident that in the presence of homoplasy Wagner trees were suboptimal solutions. In order to obtain better solutions it was necessary to do what we now know as “branch swapping”, the exchange of branches on a tree with the object of refining a previous solution. The first of such swappers, incorporated into the program PHYSYS (Farris and Mickevich, 1980), was named *branch-breaking* and later on referred to as *tree bisection and reconnection* (Swofford and Olsen, 1990). In subsequent years, *nearest-neighbor interchanges* (known as NNI), *subtree prunning and regrafting* (known as SPR; Fig. 1) and *tree bisection and reconnection* (TBR; Fig. 2) became the standard algorithms for branch swapping. These common branch-swapping algorithms (often simply referred to as *swappers*) are described in every systematics treatise (e.g. Swofford et al. 1996; Page and Holmes, 1998; Schuh, 2000; Felsenstein, 2004), and I will not explain them here. Important issues with these common swappers are the number of possible rearrangements (Allen and Steel, 2001), as well as issues of greediness (level of acceptance of a tree during the branch swapping process) that may lead to faster searches. Specific algorithms may be used for calculating tree scores more rapidly (Goloboff, 1993; Gladstein, 1997; Ronquist, 1998). Algorithmic shortcuts (e.g. Kosakovsky, Pond and Muse, 2004; Stamatakis et al. 2002) and the use of simple models to replace more complex statistical models (Stamatakis, 2005c) can also be used for calculating the likelihood of a tree more efficiently.

Due to the large number of possible trees (Felsenstein, 1978), there exists the possibility of getting stuck in local optima (Maddison, 1991). Heuristic procedures usually cope with this by building many initial trees (e.g. Wagner trees using a random addition sequence of taxa) and submitting each one of these initial trees to a branch swapping process; this is what we often refer to as *replicates* (or RAS + swapping; Goloboff, 1999).

Heuristic methods using a combination of these swapping algorithms and shortcuts can find optimal solutions for moderately sized problems (e.g. below 100 taxa for pre-aligned datasets) for which exact solutions can be calculated. More simply, for most empirical datasets that can be analyzed exhaustively, TBR will find the same solution orders of magnitude more quickly. A corollary of this is that exact methods are of little interest to most practicing systematists. Therefore, the focus of this review is the different heuristic algorithms and search strategies that aim to find heuristic solutions for optimality criterion-based phylogenetic methods. The topic of efficiency of algorithms will be also briefly discussed, at least in the context of recent improvements. This is always done in the mode of shortcuts that reduce the number of mathematical operations that need to be performed for a given action. For example, SPR branch swapping requires *t*^2^ accommodations (where *t* is the number of terminals) of clipped nodes while TBR requires *t*^3^. An impressive speedup in TNT performs quick TBR, whose execution time scales on *t*^2^ instead of *t*^3^.

## The Necessity of Refined Heuristic Procedures

Collecting phylogenetic data—especially for molecular analyses—has become easier and easier following the technological developments of the last two decades. Large data sets including several hundreds of terminal taxa are becoming common (e.g. Chase et al. 1993; Lipscomb et al. 1998; Soltis et al. 2000). A few data sets already surpass the 1,000-taxon barrier (e.g. Källersjö et al. 1998; Tehler et al. 2003; Hibbett et al. 2005; Williams and Smith, 2006; McMahon and Sanderson, 2006), and some bacterial datasets go beyond the 10,000-taxon barrier (e.g. Ribosomal Database Project [http://rdp.cme.msu.edu/index.jsp]). Under recent broad funding initiatives, such as the US NSF *Assembling the Tree of Life* project, several large data sets (ranging between 500 and 10,000 taxa) will be available for analysis in a matter of years. In order to analyze these data sets, researchers can follow two main strategies: (1) the analysis of the complete data sets, or (2) conduct separate analyses and combine the solutions using a supertree technique (e.g. Sanderson et al. 1998; Bininda-Emonds et al. 2002; Driskell et al. 2004). The focus of this article is the analysis of complete data sets (the supermatrix approach) and therefore we will not discuss supertree techniques or their implications (for details on algorithmic implications and supertree techniques see a recent review by Goloboff, 2005). Data sets with large numbers of taxa, however, are very hard to analyze using the traditional algorithms and will require further developments.

Students and researchers often wonder how many replicates they need to run in order to conduct a thorough tree search on a given data set, or what heuristic algorithms will yield the best and fastest result. The answers to these questions are not trivial since tree searches depend not only on the number of terminals, but also on the structure of the analyzed data. The only good recipe one can receive for performing tree searches would be data set-specific, and therefore only by understanding the algorithms involved will the researcher be able to design a proper search strategy. Nonetheless, certain techniques tend to work better than others for most data sets, and this knowledge can be used as a starting point for experimenting and fine-tuning the algorithms. For example, genetic algorithms are better employed after a population of near-optimal candidate trees has been generated using hill-climbing or simulated annealing algorithms.

The intention of this article is twofold. On the one hand, I will review several algorithms and search strategies that can be applied to a set of data by the investigators. The algorithms and strategies discussed here apply in general to both parsimony and maximum likelihood searches, although some techniques may have to be adapted to the different methodologies. Not all strategies or algorithms are available in all software packages, but one cautions the reader that lack of implementation should not be a reason for ignoring or dismissing more efficient strategies. The fact that our favorite software package does not incorporate algorithm X is not a scientific reason for performing a deficient tree search and fail to analyze the data properly. As the second objective of this review, I hope that it serves to stimulate software developers to implement some important recent algorithmic developments and search strategies into their packages.

## Heuristic Methods and Efficient Tree Searches—Algorithms

While discussing different algorithms, we will be referring to specific software of general use by systematists. Some of the packages more often referred to are (alphabetically): PAUP* (Swofford, 2002), POY (Wheeler et al. 2002; Varón et al. 2007), RAxML-VI-HPC (Stamatakis, 2006; Stamatakis et al. 2005) and TNT (Goloboff et al. 2003). Other software will also be referred to for specific algorithms or implementations. By no means I will attempt to refer to all software packages available in the market, and of course I restrict myself to those that I know best, or more importantly, to those that incorporate new or fast algorithms to illustrate the points of this review.

### Hill-climbing (“traditional”) algorithms

Traditional algorithms employed for tree searches combine fast methods of tree building followed by NNI, SPR or/and TBR branch swapping. The first step requires building a tree or selecting a random tree, although the latter option is generally not very efficient. Typically, the initial tree can be obtained by some sort of sequential addition of taxa, where one starts with a tree with three taxa and the following taxa are added sequentially. One could follow alphabetical order, some sort of distance measure, or by trying the new taxon in all possible branches and selecting the optimal position for the taxon added (the Wagner algorithm). This is called stepwise addition (Swofford et al. 1996), sequential addition (Felsenstein, 2004), or build (or multibuild if the addition of taxa is done in parallel) (Wheeler et al. 2002) by different authors. Other algorithms, such as star-decomposition (e.g. neighbor-joining; Saitou and Nei, 1987) have little applicability in optimality criterion-based methods.

The tree obtained after the initial build is generally used for subsequent refinement, as done by SPR and TBR. It is assumed that by using many starting trees where the order of addition of taxa has been randomized (varying the seed number of the software; most packages use a defined seed for initializing the pseudorandom generator of numbers by default) the chance of avoiding local optima increases. Therefore, any sensible search requires repeating the initial tree building step followed by branch swapping a number of times. This strategy is what it has been referred to as *conventional search methods* (Davis et al. 2005) or *traditional searches* (Goloboff et al. 2003).

Obviously the different swappers tried will affect the tree searches because the simplest ones will attempt fewer taxon arrangements than the more complex ones. In a typical search, NNI will examine 2(*t* – 3) neighbors for *t* taxa, while SPR branch swapping requires *t*^2^ accommodations of clipped nodes and TBR requires *t*^3^. As mentioned above TNT performs quick TBR, whose execution time grows *t*^2^ instead of *t*^3^. In some maximum likelihood implementations, SPR often works with a more restricted neighborhood of promising moves to avoid large numbers of intensive calculations (Hordijk and Gascuel, 2005).

In the case of static homology (morphological matrices or pre-aligned sequence data) in parsimony analyses, building the initial tree by the Wagner algorithm can be very quick. Given that Wagner trees can be obtained quickly one may want to build multiple Wagner trees and continue only with a set of the best Wagner trees to the subsequent branch swapping steps.

In the case of ML implementations, different programs do different things. Default settings in PAUP* lead to re-optimizing every branch multiple times during Wagner addition of taxa. Programs such as RAxML-VI do not reoptimize every branch during Wagner addition, and use parsimony for this step, allowing adding thousands of taxa in a matter of minutes to hours (based on a 25,000 taxon data set; Stamanakis, pers. comm.). This is also an option in POY v. 4 (Varón et al. 2007). Obviously, most of the time spent in ML calculations is employed in branch length optimization.

The basic swappers for the hill-climbing algorithms are currently available in most software packages. Some programs simply allow choosing either SPR or TBR, while others allow searching by using first SPR and continuing with TBR, making searches more efficient. The reason for this higher efficiency is clear, SPR is much faster than TBR (although it obviously depends on the implementation), so it reaches a given solution in less time than TBR does. Only after SPR cannot find a better solution, TBR continues with the search, speeding the global search time for each replicate. This is illustrated by the following example where a metazoan data set (Giribet and Wheeler, 1999) is analyzed with TNT. The analyses all start with the same random seed (seed = 1), to make results comparable, and consist of 100 replicates using (a) the Wagner algorithm, (b) Wagner + SPR, and (c) Wagner + TBR. In all cases the number of trees to swap per replicate is limited to 10. All PC analyses were performed on a Dell Precision 340, Pentium IV (2.00 GHz) with 512 Mb of RAM.

**Table:** 

**Algorithm**	**steps**	**trees exam**	**sec**
TNT-Wagner	7,078		1.39
TNT-SPR	7,030	1.6 × 10^8^	82
TNT-TBR	7,029	7.4 × 10^8^	129

The same analyses were repeated for the ‘Zilla’ data set (see Goloboff, 1999).

**Table:** 

**Algorithm**	**steps**	**trees exam**	**sec**
TNT-Wagner	16,376		9.33
TNT-SPR	16,229	1.9 × 10^8^	44.89
TNT-TBR	16,227	2.0 × 10^8^	61.19

As expected, tree length decreases dramatically when using branch swapping algorithms with respect to the Wagner tree. This Wagner tree could be in some sense comparable to a tree obtained under a method not dependent on an optimality criterion, such as neighbor-joining. Tree length decreases—to the expense of computation time—when using more complex swappers. However, in these cases the difference in execution time between SPR and TBR is not spectacular (increase of ca. 50%) due to the efficiency of the TBR algorithm. Certainly the use of shortcuts for completing a round of branch swapping has an influence in the results. When the same ‘Zilla’ data set is analyzed with a different random seed (seed = 2) the results are rather different, with a 5-step improvement between SPR and TNR branch swapping.

**Table:** 

**Algorithm**	**steps**	**trees exam**	**sec**
TNT-Wagner	16,399		9.17
TNT-SPR	16,233	1.9 × 10^8^	43.92
TNT-TBR	16,228	2.7 × 10^8^	78.63

Many programs can go further in the utilization of basic swappers, and allow using SPR, TBR, or a combination of both by using SPR first until no improvement is achieved and continuing with TBR. It also allows determining the number of trees to be retained in SPR and TBR independently, even if both algorithms are used in conjunction. POY allows specifying not only the total number of trees to retain for a given search, but also the number of trees to retain during the SPR and TBR steps. This allows the investigator to conduct more thorough searches by for example building a “quick” tree followed by fast SPR and swapping with TBR from then on.

With the availability of TBR as the most efficient swapper, and the concern of being able to escape local optima, some authors opted for storing trees up to n steps longer than the most parsimonious trees, as done with the command *jump n* of Nona (Whiting et al. 1997; Giribet and Ribera, 1998). However, this command was not very efficient and it wasted enormous amounts of time swapping on large pools of suboptimal trees. Most likely computation time could have been used more efficiently by completing more replicates.

These are basic principles to use the most common swappers efficiently. To learn the specific commands from each software package, the investigator should note the command descriptions. Also, the effect of using different size tree buffers has been explored in detail in Davis et al. (2005).

### Optimizing branch lengths in ML analyses

While the only parameters that matter under the parsimony criterion are the topology and cost of the tree, other methods also take into account branch lengths and model parameters (optimization of parameters in complex models like GTR + Γ + I can sometimes take up to 20% of total run time; A. Stamatakis, pers. comm. 2007). An obvious issue when conducting searches under the maximum likelihood optimality criterion is the time spent optimizing such branch lengths. Typically, branches are optimized one at a time in a strictly hill climbing iterative fashion where all non-optimal branch lengths are discarded. Branch lengths are often optimized using the Newton-Raphson method (Swofford et al. 1996), and for example PAUP* allows multiple options for performance tuning by controlling, among other parameters, the number of smoothing passes and the threshold at which improvement in total likelihood score is not accepted. A similar procedure is used in fastDNAml (Olsen et al. 1994) and POY, where each generated topology is evaluated by exhaustive branch length optimizations. If one of those alternative topologies improves the likelihood score is updated accordingly and once again all possible subtrees are rearranged. This process of rearrangement steps is repeated until no better topology is found.

While traditional likelihood programs optimize all branch lengths whenever a rearrangement is tried, faster algorithms introduce important speedups. RAxML-III (also RAxML-II and previous versions) only optimizes the three local branches adjacent to the insertion point, and can do this by the slower Newton-Raphson method or via a faster analytical method before computing its new likelihood value (Stamatakis et al. 2005). Since the likelihood of the tree strongly depends on the topology per se, this fast pre-scoring can be used to establish a small list of potential alternative trees, which are very likely to improve the score of the best tree. Another alternative is to optimize topology and branch length simultaneously, as it is done with the PHYML package (Guindon and Gascuel, 2003).

Although branch length optimization is a fundamental issue in likelihood calculations, I will not dedicate more space to it and I will rather concentrate in those aspects of tree searches that are of general application to all methods based on optimality criteria—topological calculations.

### Accelerating searches using ratcheting techniques

One of the most innovative search strategies using (then) available swappers is the ratcheting technique developed by Nixon (1999) and implemented in software such as Winclada (Nixon, 2002)—which uses Nona (Goloboff, 1994) as a subsidiary program to do the actual searches—TNT, or POY. It can also be used in PAUP* with the subsidiary programs PRAP (Müller, 2004a, b) or PAUPRat (Sikes and Lewis, 2001). The ratcheting strategy relies on iterative perturbations of the tree landscape in order to escape from local optima much faster. This is done by generating a tree via standard algorithms (e.g. Wagner tree + TBR) until the tree cannot be improved (it is recommended to use small tree buffers). The weight of a certain proportion of characters is altered (different implementations use different proportion of reweighted characters and different weights) and the altered matrix is used to continue swapping on the previous tree until no further improvement is made. The weights then revert to the original ones and branch swapping continues. The whole process is repeated a given number of times for each original replicate. This strategy allows escaping from local optima much more quickly than simple replicates of Wagner + TBR do. Since its description, the ratchet has been employed in numerous studies that deal with complicated data sets (e.g. Giribet and Wheeler, 1999; Goloboff, 1999; Nixon, 1999; Quicke et al. 2001).

As discussed by Nixon (1999), ratcheting techniques do not need to be restricted to parsimony, hence a similar strategy has been extended to likelihood tree searches (Vos, 2003), although not implemented in any software package. A recent experimental implementation of a ratchet in RAxML did not show any improvements compared to the standard hill-climbing algorithm (A. Stamatakis, pers. comm. 2007).

### Genetic algorithms

A new family of algorithms, based on the principle of recombination among trees, have been described by several authors (Matsuda, 1996; Lewis, 1998; Moilanen, 1999, 2001; Goloboff, 1999; Zwickl, 2006). The principle of this family of methods is to extend branch swapping of basic algorithms such as SPR and TBR to exchanging branches among different trees. So the basic algorithms (SPR and TBR) could be described as intra-tree branch swapping algorithms while the genetic algorithms refer to inter-tree branch swapping.

The most common and efficiently implemented genetic algorithm is Goloboff’s tree fusing (TF) algorithm (Goloboff, 1999, 2002), currently implemented in TNT and POY. The method compares different trees and exchanges compatible clades among them. Tree fusing improves on Moilanen’s algorithm, which exchanged one randomly chosen subclade at a time, placing it in a ramdomly chosen position. Goloboff’s TF exchanges all the groups with a certain number of taxa that can be specified and that is found in the consensus of both trees. The best result is obtained when multiple trees are available to exchange clades. Tree fusing has been used in several recent studies that deal with large or complicated data sets (Okusu et al. 2003; Edgecombe and Giribet, 2004; Giribet et al. 2004; Lindgren et al. 2004; Wheeler et al. 2004; Giribet et al. 2005). TF comes in different flavors and trees are fused in different ways, exchanging subtrees in one or in both directions and saving a different number of trees. TF, as implemented in TNT, is extremely fast and allows reaching a “nearly-optimal” solution in truly short execution times, but it generally does not suffice to find an optimal solution without the aid of other algorithms (Goloboff, 1999). Other genetic algorithm implementations include the Cooperative Rec-I-DCM3 (Williams and Smith, 2006), which have shown good performance behavior when the number of cooperative trees is not too small, although this is not available to the public and therefore may be of little value to the community.

Maximum likelihood implementations include the *metapopulation genetic algorithm* found in METAPIGA (Lemmon and Milinkovitch, 2002) and the GAML algorithm (Lewis, 1998; Brauer et al. 2002). The application of the genetic algorithms family goes beyond the ones described here or in the original papers; it efficiently allows to incorporate results from previous analyses for the population of trees where exchanges are to be performed (see below).

### Divide and conquer algorithms

Another interesting set of algorithms are the “divide and conquer” family of algorithms, which aim at reducing the dimension of the solution space by restricting a given problem to subsets of smaller problems. A primitive divide and conquer method is the quartet technique (Strimmer and von Haeseler, 1996), which divides the data in 4-taxon trees, although this technique has been shown to be a poor estimator of phylogeny.

More sophisticated divide and conquer strategies are illustrated by two specific algorithms, Goloboff’s sectorial searches (SS) (Goloboff, 1999, 2002) and the disccovering family of methods (DCMs) (Nakhleh et al. 2001; Roshan et al. 2004). SS needs a tree as a starting point, and different sectors of the tree are reanalyzed separately; if a better configuration is found, the new sector replaces the old one on the novel tree. These reduced data sets can be analyzed quickly. Sectors can be selected in different ways, randomly or based on consensus (Goloboff, 1999).

The DCM family of methods also analyzes sectors of a tree, but in this case it does it by contracting the nodes in the remainder of the tree rebuilding a new matrix. The logic behind these methods is that it is much harder to achieve an optimal configuration for the entire tree than it is for smaller sectors of this tree. DCM therefore differs from SS in that SS use both OTUs and HTUs while DCM only uses OTUs.

Ota and Li (2000), Ota and Li (2001) have also developed a type of “divide and conquer” method that combines neighbor-joining support and maximum likelihood calculations.

### Simulated annealing methods

In hard optimization problems, such as tree searches, accepting suboptimal solutions with a certain probability is generally known as simulated annealing (Kirkpatrick et al. 1983). Earlier implementations of simulated annealing methods in Metro (a program formerly included in PHYLIP) performed poorly. Currently, Goloboff’s tree-drifting (DFT) algorithm (Goloboff, 1999, 2002) is implemented in TNT and in POY, and other less used implementations for parsimony analysis also exist (Barker, 2004). DFT determines the acceptability of a tree by using both its raw length difference and the relative fit difference (RFD) (Goloboff and Farris, 2001). The algorithm is based on doing rounds of TBR, alternatively accepting only optimal trees or optimal and suboptimal trees. Then, as in the ratchet, the suboptimal trees are discarded, until a new round of drifting starts and the exercise is performed a number of times. Tree drifting is almost as effective as the ratchet at finding optimal trees (Goloboff, 2002), with small differences depending on implementation.

The first application of a simulated annealing algorithm to maximum likelihood analyses was presented by Salter and Pearl (2001), and more recently an elegant implementation was added to the RAxML family of programs. RAxML-SA (Randomized Axelerated maximum Likelihood with Simulated Annealing) combines hill-climbing techniques with “backward steps” to slightly improve scores of final trees when compared to those available in its predecessors (Stamatakis, 2005a). However, this strategy has been abandoned in current releases due to its very slow inference time (A. Stamatakis, pers. comm. 2007).

## Heuristic Methods and Efficient Tree Searches—Strategies

In the paragraphs above, common algorithms and several simple search strategies that can be used in order to conduct more or less efficient tree searches are reviewed. In this section, the focus is on a set of search strategies beyond those simple ones. In fact, a wise utilization of tree buffer size, number of replications, constrained searches, or the methods for altering the tree landscape will determine the efficiency of tree searches. The focus of this section centers in two main strategies, (1) combination of algorithms described in the previous one, and (2) the intelligent or “driven” searches. A review of these aspects can be found in Goloboff (2002).

### Accelerating searches using traditional algorithms: tree buffers

In addition to the choice of swapping algorithm, the number of trees to be retained per replicate plays a fundamental role in the efficiency of tree searches (Giribet and Wheeler, 1999; Davis et al. 2005). Davis et al. (2005) discussed in detail the effects of increasing the amount of swapping per replicate (increasing the number of trees retained per replicate from 1 to 5,000), and not surprisingly they found that the more trees are retained, the more times minimum tree length is found. But this is done at the expense of computation time. However, they conclude that the limit of these conventional analyses lays for matrices with up to 500–700 terminals.

It is beyond the objective of this article to discuss specific commands for specifying the size of tree buffers in the different software packages. As a general rule, tree buffers can be specified globally for an entire search, per replicate, or even for the different steps of a given search (e.g. specifying different maxtrees for the SPR step, TBR step, et cetera).

To illustrate the issue of the number of trees to be retained per replicate, the metazoan data set described above was analyzed under parsimony using TNT in two ways. First, I analyzed the data in the same conditions listed above, but retaining 100 trees per replicate instead of 10 trees per replicate. Second, I ran the same data during 10 minutes (a) setting the number of trees per replicate to 10,000 or (b) retaining 10 trees per replicate. The first analysis completed 6 replicates (5 × 10^9^ trees examined) and found a minimum tree length of 7,031 steps. The analysis retaining 10 trees per replicate allowed completion of 739 replicates in the same amount of time, examining an equivalent number of trees, but resulted on trees 3 steps shorter and radically different phylogenetic hypothesis with respect to Ecdysozoa. Clearly, the second strategy, by investing less effort on each replicate, allowed exploring a broader tree space, not wasting time swapping on trees from the same suboptimal island. In addition, collapsing rules may also have an important effect on execution times.

### Accelerating searches using traditional algorithms: constraints

Tree searches are complex because the number of possible trees grows exponentially with the number of terminal taxa included in the analyses. An easy way to ameliorate the problem of the large number of trees is by decreasing the effective number of terminals in an analysis. However, since taxon sampling has been demonstrated to be a key factor for phylogenetic accuracy (e.g. Wheeler, 1992; Hillis, 1996; Giribet and Carranza, 1999; Pollock et al. 2002; Zwickl and Hillis, 2002; Hedtke et al. 2006), decreasing the real number of terminals is not a good idea, unless of course they are redundant.

An easy way to decrease the effective number of nodes to be swapped without decreasing the real number of taxa (and character states observed within those taxa) is by using constraints during tree searches. Constraint searches are often used for exploring topologies or testing hypotheses and optimizing parameters. Constraints can be specified in some available software packages such as GARLI, Nona, PAUP, POY, RAxML, or TNT, however, the use of constraints needs to be carefully designed. Of course, the use of constraints can have a direct effect on the final topology if the nodes being constrained were not present in the true tree. For example, Giribet and Ribera (2000) used jackknife frequencies above 95% as a constraint for a subsequent search in order to speed up the analyses. As resampling techniques for methods based on optimality criteria can take an enormous amount of time, other strategies could be used, such as using some high threshold (100%) for neighbor-joining bootstrapping. This trivial strategy has been seldom used despite its logical speedup of analyses, and it may depend on the development of techniques for quick consensus estimation (Goloboff and Farris, 2001). This technique is related to divide and conquer techniques.

### Accelerating searches using traditional algorithms: previous searches

Another strategy that can be employed is the use of trees obtained during previous analyses, not necessarily by the investigator. These trees could be obtained from a “tree database”. Systematists tend to build upon previous work to further their research so not all the information utilized in an analysis needs to be generated *de novo*. The use of molecular data from GenBank and other databases is commonplace in molecular systematics. The same applies to morphological systematics, where researchers distill previously published morphological work and often refine, expand or merge previous matrices to come up with a more perfected hypothesis. This makes sense because generating sequence data or morphological observations is not a trivial process, and if other investigators have invested resources and time to generate those observations, why should we generate them again? In an ideal world with error-free databases and data matrices, generating data *de novo* at every step would be insensible.

Generating trees also costs time and money (Mecham et al. 2006). There are several published articles praising on the computation effort invested into generating trees and the use of computer clusters for phylogenetic analyses is growing exponentially because investigators need more sophisticated analyses to avoid problems of local optima when analyzing large data sets. However, there is little use of previously generated phylogenetic hypotheses as a starting point for a new phylogenetic analysis. Here, recycling of previous analyses is proposed as starting points for new phylogenetic analysis, even if the previous analyses contain fewer taxa than the newly analyzed data sets. This strategy has been recently called “jumpstarting phylogenetics” (Mecham et al. 2006).

Most programs allow initiating the swapping process with an input tree, obtained randomly or by reading it from a tree file. This strategy saves time, especially in the case of traditional maximum likelihood analyses because the initial tree for a parsimony search is calculated much more rapidly than building a tree for a maximum likelihood tree. This is so because in addition to topologies, the likelihood algorithm evaluates branch lengths (Swofford et al. 1996; Goloboff, 2003). Some programs have therefore incorporated this strategy by initiating the likelihood searches from a tree generated via parsimony or some clustering method, since these trees are often more optimal under the likelihood criterion than a random or a Wagner tree are (Guindon and Gascuel, 2003; Stamatakis et al. 2005). For example, RAxML-VI can start with a reduced tree not containing all taxa of an extended data set. Remaining taxa will be added by Wagner parsimony addition and global optimizations will then be performed via maximum likelihood.

The use of previously generated trees is also important when applying complex phylogenetic algorithms such as iterative pass optimization (Wheeler, 2003), which uses three nodes instead of two for a given optimization problem and therefore is able to find more parsimonious solutions than those of direct optimization at the expense of computation time. The use of direct optimization trees as a source for continuing with the iterative pass calculations is commonplace (Wheeler, 2003; Faivovich et al. 2004; Smith and Wheeler, 2004; Giribet and Edgecombe, 2006).

**Figure 3.**
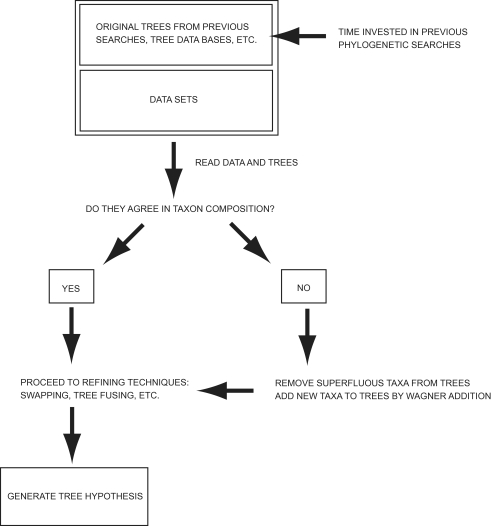
Flowchart of the pre-processed search technique described here.

However, except in these cases, few analyses use results from previous searches to continue estimating phylogenetic hypotheses. I propose here to utilize results from previous searches containing fewer taxa using the same optimality criterion and model as a starting point, continue adding taxa with a random seed, and repeat this for a given number of replicates. This will ensure that most nodes, which had been optimized in a similar search in a previous analysis (or series of analyses) and therefore remain at a near-optimal configuration, will give structure to the analysis. In order to conduct this kind of analysis, the phylogenetic software would need to be able to read a tree with fewer taxa than the existing data set, and continue with a Wagner addition for the taxa not included in the original tree. This requires reading tree files with fewer taxa than the data matrices stored in memory, and this is done by comparing the tree file to a file that contains a list of all the terminals to be analyzed^2^. While the strategy discussed in the previous section could be called *constrained searches*, the strategy described here could be referred to as *pre-processed searches*. Although this technique has been in use for a number of years by POY users (since the incorporation of the command –terminalsfile), it has also been described in a more general context recently as a mode of “jumpstarting” phylogenetic analyses (Mecham et al. 2006) (see flowchart for a preprocessed search in Fig. 3).

Pre-processed searches are also very useful in cases where new characters are added to the previous runs, or in the common case of correcting errors or adding missing data to a prior analysis. The change of a few characters in a large analysis may not pose a dramatic change to the previous trees and therefore it may not be worth redoing an entire analysis. Therefore using previous results as a starting point for the search is a wise way to proceed.

### Accelerating searches using genetic algorithms and pre-processed searches

A special strategy involving genetic algorithms has also been designed by providing a population of trees obtained under different analytical parameters via a sensitivity analysis (Wheeler, 1995; Giribet, 2003) and submitting those trees to tree fusing (Wheeler et al. 2004). This strategy has been termed *sensitivity analysis tree fusing* or SATF (D’Haese, 2003; Wheeler et al. 2005) and has been proven to increase both analytical speed and efficiency for medium to large data sets (Boyer et al. 2005; Sørensen et al. 2005; Giribet et al. 2006). The possibilities of SATF for maximum likelihood analyses seem extremely promising.

Another strategy consists in generating an initial population of trees via jackknifing (by using the command –jackstart in POY), and proceeding to tree fusing. Other methods, such as bootstrapping, should give similar results. In some cases, trees generated through a sensitivity analysis and jack-knife trees could be combined to constitute the population of trees to be fused by the genetic algorithms.

### Combining algorithms

While most of the algorithms described above are efficient under a range of conditions, they often perform better when combined. Goloboff (1999) clearly showed how certain algorithms decrease tree length rapidly but others may be required to actually find the optimal solution (in his example data sets). Combining these algorithms with intelligent search strategies (ratcheting, drivers, constraint searches, pre-processed searches, and the like) is likely to be a more efficient phylogenetic strategy, but unfortunately the entire family of algorithms and strategies is only found in the parsimony search program TNT (see a review of the program in Giribet, 2005), and a subset of these are incorporated in POY (see Wheeler et al. 2006). Ratcheting and tree fusing (Lewis, 1998; Lemmon and Milinkovitch, 2002; Vos, 2003; Wheeler et al. 2005) and simulated annealing (Stamatakis, 2005a) are also incorporated in likelihood-based software, but combinations of the different “new technology” algorithms are not available in probabilistic approaches.

While current software has improved tremendously in the amount of taxa that can be handled, for both parsimony and maximum likelihood analyses (Goloboff et al. 2003; Roshan et al. 2004; Stamatakis, 2005a, b; Stamatakis et al. 2005), still the amount of taxa that can be analyzed in reasonable amounts of time is a limiting factor. For example, a parallel version of TNT finds optimal solutions in the *zilla* data set in a matter of seconds and the sequential version of RAxML-VI was able to finish a maximum likelihood replicate for 25,000 taxa in 4–5 days (Stamanakis pers. comm. 2007).

The algorithms described above can obviously be combined in different ways. From an experimental point of view and working with large data sets in TNT, the best results are obtained when multiple replicates of Wagner trees are swapped using TBR and followed by sectorial searches, then drifting or ratcheting, followed by tree fusing (Goloboff, 1999, 2002), at least in the case of the *zilla* data set. Fine-tuning of each algorithm is necessary for best performance and other strategies could obviously be tried in other software programs. What seems clear is that multipe rounds of Wagner addition to start the analyses are fundamental, as it is finishing them with tree fusing—at least in the case of complex data sets. The algorithms used in between (ratcheting, simulated annealing, sectorial searches) may yield optimal results depending on the data. In the case of POY, the combination of multiple rounds of ratcheting and tree fusing is commonly used, but little performance testing has actually been done.

Another factor that has received little attention in the literature is sequence length requirements for accurate reconstruction of phylogenies (e.g. Moret et al. 2002). This aspect will not be discussed further in this review.

## When is a Search Good Enough?

One of the main issues when applying heuristic algorithms to tree search is defining a stopping rule. Historically, researchers defined the number of Wagner random addition replicates a priori, and in the best case, if the number of best solutions was a small fraction of the total replicates, the search would be extended to more replicates. In other cases, searches were limited by execution time. However, none of these methods allows for a sound evaluation of the results of the heuristic search in terms of convergence and reliability of results. An alternative to this is the use of a specified set of stopping rules, the most well defined are called “driven searches”.

### Driven searches

The term *Driven Searches* is used in the computer program TNT to designate a series of intelligent searches where the user does not define the number of replicates to be performed, but instead uses search strategies that continue searching until achieving a certain goal (Goloboff, 1999, 2002; Goloboff et al. 2003).

One of the first analyses using some sort of driven search is that of Giribet et al. (2001). This study used POY in a way that the authors could define a simple search strategy such that it would do a number of replicates (100 in that case), but it would stop the search once minimal tree length had been found three times after having performed at least 10 full replicates (commands –replicates^3^ 1000 –stopat 3 –minstop 10). However, it has been shown that results often need more replicates to converge on a stable consensus (D. Pol pers. comm. 2005).

More interesting driven searches are those implemented in TNT. The most obvious driver is to specify a fixed number of times that a minimum tree length has to be found during the search; for example one can ask to keep searching and then stop after minimum tree length (defined as the minimum length the program is able to find) is hit 5 times. This is based on the notion that convergence in a solution may be a desirable property when using heuristics.

Other more sophisticated drivers involve consensus techniques, where one searches until minimum tree length is found a certain number of times and then a consensus is estimated. A second round of searching starts and a new consensus is generated and compared to the previous one, and so on until the consensus stabilizes. The number of hits to minimum tree length as well as the times that the consensus is stable, or can be defined by the user. This method works extremely well for data sets with thousands or millions of equally parsimonious trees, as is typical of some morphological data sets with many missing data. The use of such drivers allows achieving a stable consensus after finding just a few trees, without the necessity of expending computation resources in obtaining all the MPTs, which will be collapsed anyway. The drivers are thus another important component of tree searches, although perhaps not as well known as the incorporation of tree searching algorithms. Consensus techniques have required advances on quick collapsing methods (Goloboff and Farris, 2001) to make drivers a viable option.

Slightly related to the drivers is the “–fitchtrees *n*” command in POY (Wheeler et al. 2006). This is based on an unpublished algorithm proposed by W. Fitch, and affects the behavior of the tree buffers (e.g. –holdmaxtrees *n*) by storing the most diverse set of trees the program can find instead of the first *n* trees specified in the buffer. This should contribute towards the goal of achieving a stable consensus.

## Conclusion

Phylogenetic methods based on optimality criteria are highly desirable for their inherent properties, but slow when compared to other methods of tree construction. Traditionally, researchers have been limited to exploring tree space by using multiple replicates of Wagner addition followed by SPR or/and TBR branch swapping but these methods have been shown to be insufficient for large data sets or even for small data sets with a rugged tree space. Other strategies not yet widely used, such as constraint searches or the pre-processed search technique here proposed could drastically decrease computation time. But major progress comes from recent new algorithms such as the ratchet (Nixon, 1999; Vos, 2003), genetic algorithms (Lewis, 1998; Goloboff, 1999; Moilanen, 1999, 2001), divide and conquer algorithms (Goloboff, 1999; Nakhleh et al. 2001; Roshan et al. 2004), and simulated annealing methods (Goloboff, 1999; Stamatakis, 2005a). Combination of clever search strategies, such as driven searches, and new algorithms has drastically increased the number of taxa that can be analyzed in reasonable amounts of time. Finally, the addition of parallelism to the developing toolkit of the practicing systematist has also had a positive impact, allowing the analysis of complicated data sets, especially for the computationally intensive direct optimization methods which consist of several nested NP-complete problems, and holds important promises for the future as more software is currently being developed to work in parallel.

## Footnotes

1 For the purpose of this discussion I will avoid other methods derived from the purely optimality criterion-based methods, such as Bayesian phylogenetics or parsimony jackknifing, because they are based on repeating “quick-and-dirty” analyses a large number of times.

2 This option of the computer program POY has demonstrated to be extremely useful in many situations, especially for selecting subsets of taxa from a master file that contains a much larger number of terminals. Important applications of this “terminals file” are for databasing and for combining partitions with different taxa in simultaneous (concatenated) analyses. The same way that the “terminals file” adds missing taxa to a tree (or to a data matrix, by including an “all missing data” terminal), it also can serve to remove taxa not specified in the “terminals file” (both, from input trees or from data matrices). A nice possibility of such files could be the use of synonyms for terminal names; e.g., Drosophilamelanogaster = Dmelanogaster = melano1, etc. would allow to use either name in different data sets or input trees, simplifying the concatenation of data from different sources.

3 In the original study of Giribet et al. (2001) the command “–random 100” was used instead of “–replicates 100”, but this commands are identical, involving only a name in the command.

## References

[b1-ebo-03-341] Allen BL, Steel M (2001). Subtree transfer operations and their induced metrics on evolutionary trees. Annals of Combinatorics.

[b2-ebo-03-341] Barker D (2004). LVB: Parsimony and simulated annealing in the search for phylogenetic trees. Bioinformatics.

[b3-ebo-03-341] Bininda-Emonds OR, Gittleman JL, Steel MA (2002). The (super)Tree of Life: Procedures, problems, and prospects. Annual Review of Ecology and Systematics.

[b4-ebo-03-341] Boyer SL, Karaman I, Giribet G (2005). The genus Cyphophthalmus (Arachnida, Opiliones, Cyphophthalmi) in Europe: a phylogenetic approach to Balkan Peninsula biogeography. Molecular Phylogenetics and Evolution.

[b5-ebo-03-341] Brauer MJ, Holder MT, Dries LA, Zwickl DJ, Lewis PO, Hillis DM (2002). Genetic algorithms and parallel processing in maximum-likelihood phylogeny inference. Molecular Biology and Evolution.

[b6-ebo-03-341] Chase MW, Soltis DE, Olmstead RG, Morgan D, Les DH, Mishler BD, Duvall MR, Price RA, Hills HG, Qiu Y-L, Kron KA, Rettig JH, Conti E, Palmer JD, Manhart JR, Sytsma KJ, Michaels HJ, Kress WJ, Karol KG, Clark WD, Hedrén M, Gaut BS, Jansen RK, Kim K-J, Wimpee CF, Smith JF, Furnier GR, Strauss SH, Xiang Q-Y, Plunkett GM, Soltis PS, Swensen SM, Williams SE, Gadek PA, Quinn CJ, Eguiarte LE, Golenberg E, Learn GHJ, Graham SW, Barrett SCH, Dayanandan S, Albert VA (1993). Phylogenetics of seed plants: An analysis of nucleic sequences from the plastid gene *rbc*L. Annals of the Missouri Botanical Garden.

[b7-ebo-03-341] Chor B, Tuller T (2005). Maximum likelihood of evolutionary trees: Hardness and approximation. Bioinformatics.

[b8-ebo-03-341] D’Haese CA (2003). Sensitivity analysis and tree-fusing: Faster, better. Cladistics.

[b9-ebo-03-341] Davis JI, Nixon KC, Little DP, Albert VA (2005). The limits of conventional cladistic analysis. Parsimony, phylogeny, and genomics.

[b10-ebo-03-341] Driskell AC, Ane C, Burleigh JG, McMahon MM, O’Meara BC, Sanderson MJ (2004). Prospects for building the tree of life from large sequence databases. Science.

[b11-ebo-03-341] Edgecombe GD, Giribet G (2004). Adding mitochondrial sequence data (16S rRNA and cytochrome c oxidase subunit I) to the phylogeny of centipedes (Myriapoda, Chilopoda): an analysis of morphology and four molecular loci. Journal of Zoological Systematics and Evolutionary Research.

[b12-ebo-03-341] Faivovich JP, Garcia C, Ananias F, Lanari L, Basso NG, Wheeler WC (2004). A molecular perspective on the phylogeny of the Hyla pulchella species group (Anura, Hylidae). Molecular Phylogenetics and Evolution.

[b13-ebo-03-341] Farris JS (1970). Methods for computing Wagner trees. Systematic Zoology.

[b14-ebo-03-341] Farris JS Mickevich MF 1980PHYSYS

[b15-ebo-03-341] Felsenstein J (1978). The number of evolutionary trees. Systematic Zoology.

[b16-ebo-03-341] Felsenstein J (2004). Inferring Phylogenies.

[b17-ebo-03-341] Garey MR, Graham RL, Johnson DS (1977). The complexity of computing Steiner minimal trees. SIAM Journal on Applied Mathematics.

[b18-ebo-03-341] Garey MR, Johnson DS (1977). The rectilinear Steiner tree problem is NP-complete. SIAM Journal on Applied Mathematics.

[b19-ebo-03-341] Giribet G (2003). Stability in phylogenetic formulations and its relationship to nodal support. Systematic Biology.

[b20-ebo-03-341] Giribet G (2005). Book Reviews: TNT: Tree analysis using New Technology. Systematic Biology.

[b21-ebo-03-341] Giribet G, Carranza S (1999). What can 18S rDNA do for bivalve phylogeny?. Journal of Molecular Evolution.

[b22-ebo-03-341] Giribet G, Edgecombe GD (2006). Conflict between data sets and phylogeny of centipedes: an analysis based on seven genes and morphology. Proceedings: Biological Sciences.

[b23-ebo-03-341] Giribet G, Edgecombe GD, Carpenter JM, D’Haese CA, Wheeler WC (2004). Is Ellipura monophyletic? A combined analysis of basal hexapod relationships with emphasis on the origin of insects. Organisms, Diversity and Evolution.

[b24-ebo-03-341] Giribet G, Edgecombe GD, Wheeler WC (2001). Arthropod phylogeny based on eight molecular loci and morphology. Nature.

[b25-ebo-03-341] Giribet G, Okusu A, Lindgren AR, Huff SW, Schrödl M, Nishiguchi MK (2006). Evidence for a clade composed of molluscs with serially repeated structures: Monoplacophorans are related to chitons. Proceedings of the National Academy of Sciencesof the U.S.A.

[b26-ebo-03-341] Giribet G, Ribera C (1998). The position of arthropods in the animal kingdom: a search for a reliable outgroup for internal arthropod phylogeny. Molecular Phylogenetics and Evolution.

[b27-ebo-03-341] Giribet G, Ribera C (2000). A review of arthropod phylogeny: new data based on ribosomal DNA sequences and direct character optimization. Cladistics.

[b28-ebo-03-341] Giribet G Richter S Edgecombe GD Wheeler WC 2005 The position of crustaceans within the Arthropoda — evidence from nine molecular loci and morphology Koenemann S Jenner RA Crustacean Issues 16: Crustacea and Arthropod RelationshipsFestschrift for Frederick R SchramTaylor and Francis Boca Raton 307 352

[b29-ebo-03-341] Giribet G, Wheeler WC (1999). The position of arthropods in the animal kingdom: Ecdysozoa, islands, trees, and the “parsimony ratchet”. Molecular Phylogenetics and Evolution.

[b30-ebo-03-341] Gladstein D (1997). Efficient incremental character optimization. Cladistics.

[b31-ebo-03-341] Goloboff PA (1993). Character optimization and calculation of tree lengths. Cladistics.

[b32-ebo-03-341] Goloboff PA (1994). Nona, version 1.5.1. American Museum of Natural History.

[b33-ebo-03-341] Goloboff PA (1999). Analyzing large data sets in reasonable times: solutions for composite optima. Cladistics.

[b34-ebo-03-341] Goloboff PA, DeSalle R, Giribet G, Wheeler W (2002). Techniques for analyzing large data sets. Techniques in Molecular Systematics and Evolution.

[b35-ebo-03-341] Goloboff PA (2003). Parsimony, likelihood, and simplicity. Cladistics.

[b36-ebo-03-341] Goloboff PA (2005). Minority rule supertrees? MRP, Compatibility, and Minimum Flip may display the least frequent groups. Cladistics.

[b37-ebo-03-341] Goloboff PA, Farris JS (2001). Methods for quick consensus estimation. Cladistics.

[b38-ebo-03-341] Goloboff PA Farris JS Nixon K 2003 TNT: Tree analysis using New Technology. Version 1.0, version Beta test v. 0.2Program and documentation available at http://www.zmuc.dk/public/phylogeny/TNT/

[b39-ebo-03-341] Guindon S, Gascuel O (2003). A simple, fast, and accurate algorithm to estimate large phylogenies by maximum likelihood. Systematic Biology.

[b40-ebo-03-341] Hedtke SM, Townsend TM, Hillis DM (2006). Resolution of phylogenetic conflict in large data sets by increased taxon sampling. Systematic Biology.

[b41-ebo-03-341] Hendy MD, Penny D (1982). Branch and bound algorithms to determine minimal evolutionary trees. Systematic Zoology.

[b42-ebo-03-341] Hibbett DS, Nilsson RH, Snyder M, Fonseca M, Costanzo J, Shonfeld M (2005). Automated phylogenetic taxonomy: an example in the homobasidiomycetes (mushroom-forming fungi). Systematic Biology.

[b43-ebo-03-341] Hillis DM (1996). Inferring complex phylogenies. Nature.

[b44-ebo-03-341] Hordijk W, Gascuel O (2005). Improving the efficiency of SPR moves in phylogenetic tree search methods based on maximum likelihood. Bioinformatics.

[b45-ebo-03-341] Källersjö M, Farris JS, Chase MW, Bremer B, Fay MF, Humphries CJ, Petersen G, Seberg O, Bremer K (1998). Simultaneous parsimony jackknife analysis of 2538 rbcL DNA sequences reveals support for major clades of green plants, land plants, seed plants and flowering plants. Plant Systematics and Evolution.

[b46-ebo-03-341] Kirkpatrick S, Gellat C, Vecchi M (1983). Optimization by simulated annealing. Science.

[b47-ebo-03-341] Kosakovsky Pond SL, Muse SV (2004). Column sorting: rapid calculation of the phylogenetic likelihood function. Systematic Biology.

[b48-ebo-03-341] Lemmon AR, Milinkovitch MC (2002). The metapopulation genetic algorithm: An efficient solution for the problem of large phylogeny estimation. Proceedings of the National Academy of Sciences of the U.S.A.

[b49-ebo-03-341] Lewis PO (1998). A genetic algorithm for maximum-likelihood phylogeny inference using nucleotide sequence data. Molecular Biology and Evolution.

[b50-ebo-03-341] Lindgren AR, Giribet G, Nishiguchi MK (2004). A combined approach to the phylogeny of Cephalopoda (Mollusca). Cladistics.

[b51-ebo-03-341] Lipscomb DL, Farris JS, Källersjö M, Tehler A (1998). Support, ribosomal sequences and the phylogeny of the Eukaryotes. Cladistics.

[b52-ebo-03-341] McMahon MM, Sanderson MJ (2006). Phylogenetic supermatrix analysis of GenBank sequences from 2228 papilionoid legumes. Systematic Biology.

[b53-ebo-03-341] Maddison DR (1991). The discovery and importance of multiple islands of most-parsimonious trees. Systematic Zoology.

[b54-ebo-03-341] Matsuda H, Hunter L, Klein TE (1996). Protein phylogenetic inference using maximum likelihood with a genetic algorithm. Pacific Symposium on Biocomputing ’96.

[b55-ebo-03-341] Mecham J, Clement M, Snell Q, Freestone T, SK, Crandall K (2006). Jumpstarting phylogenetic analysis. International Journal of Bioinformatics Research and Applications.

[b56-ebo-03-341] Moilanen A (1999). Searching for most parsimonious trees with simulated evolutionary optimization. Cladistics.

[b57-ebo-03-341] Moilanen A (2001). Simulated evolutionary optimization and local search: Introduction and application to tree search. Cladistics.

[b58-ebo-03-341] Moret BM, Roshan U, Warnow T (2002). Sequence-length requirements for phylogenetic methods. Algorithms in Bioinformatics.

[b59-ebo-03-341] Müller K (2004a). PRAP—computation of Bremer support for large data sets. Molecular Phylogenetics and Evolution.

[b60-ebo-03-341] Müller K 2004b PRAP, Parsimony ratchet analyses with PAUP*, version 1.0Program and documentation available at www.nees.uni-bonn.de/downloads/PRAP/

[b61-ebo-03-341] Müller K (2006). Incorporating information from length-mutational events into phylogenetic analysis. Molecular Phylogenetics and Evolution.

[b62-ebo-03-341] Nakhleh L, Roshan U, St John K, Sun J, Warnow T (2001). Designing fast converging phylogenetic methods. Bioinformatics Sippl.

[b63-ebo-03-341] Nixon KC (1999). The Parsimony Ratchet, a new method for rapid parsimony analysis. Cladistics.

[b64-ebo-03-341] Nixon KC 2002 Winclada, v. 1.00.08Program and documentation available at www.cladistics.com

[b65-ebo-03-341] Okusu A, Schwabe E, Eernisse DJ, Giribet G (2003). Towards a phylogeny of chitons (Mollusca, Polyplacophora) based on combined analysis of five molecular loci. Organisms, Diversity and Evolution.

[b66-ebo-03-341] Olsen GJ, Matsuda H, Hagstrom R, Overbeek R (1994). FastD-NAml: a tool for construction of phylogenetic trees of DNA sequences using maximum likelihood. Comput. Appl. Biosci.

[b67-ebo-03-341] Ota S, Li WH (2000). NJML: a hybrid algorithm for the neighbor-joining and maximum-likelihood methods. Molecular Biology and Evolution.

[b68-ebo-03-341] Ota S, Li WH (2001). NJML+: an extension of the NJML method to handle protein sequence data and computer software implementation. Molecular Biology and Evolution.

[b69-ebo-03-341] Page RDM, Holmes EC (1998). Molecular evolution A phylogenetic approach.

[b70-ebo-03-341] Pollock DD, Zwickl DJ, McGuire JA, Hillis DM (2002). Increased taxon sampling is advantageous for phylogenetic inference. Systematic Biology.

[b71-ebo-03-341] Quicke DL, Taylor J, Purvis A (2001). Changing the landscape: a new strategy for estimating large phylogenies. Systematic Biology.

[b72-ebo-03-341] Ronquist F (1998). Fast Fitch-parsimony algorithms for large data sets. Cladistics.

[b73-ebo-03-341] Roshan U, Warnow T, Moret BME, Williams TL Year. Rec-I-DCM3: a fast algorithmic technique for reconstructing large phylogenetic trees.

[b74-ebo-03-341] Salter LA, Pearl DK (2001). Stochastic search strategy for estimation of maximum likelihood phylogenetic trees. Systematic Biology.

[b75-ebo-03-341] Saitou N, Nei M (1987). The neighbor-joining method: a new method for reconstructing phylogenetic trees. Molecular Biology and Evolution.

[b76-ebo-03-341] Sanderson MJ, Kim J (2000). Parametric phylogenetics?. Systematic Biology.

[b77-ebo-03-341] Sanderson MJ, Purvis A, Henze C (1998). Phylogentic supertrees: assembling the trees of life. Trends in Ecology and Evolution.

[b78-ebo-03-341] Schuh RT (2000). Biological systematics Principles and applications.

[b79-ebo-03-341] Sikes DS, Lewis PO (2001). beta software, version 1 PAUPRat: PAUP implementation of the parsimony ratchet Distributed by the authors. Department of Ecology and Evolutionary Biology, University of Connecticut.

[b80-ebo-03-341] Smith WL, Wheeler WC (2004). Polyphyly of the mail-cheeked fishes (Teleostei: Scorpaeniformes): evidence from mitochondrial and nuclear sequence data. Molecular Phylogenetics and Evolution.

[b81-ebo-03-341] Soltis DE, Soltis PS, Chase MW, Mort ME, Albach DC, Zanis M, Savolainen V, Hahn WH, Hoot SB, Fay MF, Axtell M, Swensen SM, Prince LM, Kress WJ, Nixon KC, Farris JS (2000). Angiosperm phylogeny inferred from 18S rDNA, *rbc*L, and *atp*B sequences. Botanical Journal of the Linnean Society.

[b82-ebo-03-341] Sørensen MV, Sterrer W, Giribet G (2005). Gnathostomulid phylogeny inferred from a combined approach of four molecular loci and morphology. Cladistics.

[b83-ebo-03-341] Stamatakis A (2005a). An efficient program for phylogenetic inference using simulated annealing.

[b84-ebo-03-341] Stamatakis A 2005bRAxML-VI. Software and documentation available at www.ics.forth.gr/~stamatak

[b85-ebo-03-341] Stamatakis A (2005c). Phylogenetic models of rate heterogeneity: a high performance computing perspective. Proceedings of IPDPS 2006, Rhodos, Greece.

[b86-ebo-03-341] Stamatakis A (2006). AxML-VI-HPC: maximum likelihood-based phylogenetic analyses with thousands of taxa and mixed models. Bioinformatics.

[b87-ebo-03-341] Stamatakis AP, Ludwig T, Meier H (2005). RAxML-III: a fast program for maximum likelihood-based inference of large phylogenetic trees. Bioinformatics.

[b88-ebo-03-341] Stamatakis AP, Ludwig T, Meier H, Wolf MJ (2002). Acccelerating parallel maximum likelihood-based phylogenetic tree calculations using subtree equality vectors. Proceedings of 15th IEEE/ACM Supercomputing Conference (SC2002).

[b89-ebo-03-341] Strimmer K, von Haeseler A (1996). Quartet puzzling: a quartet maximum-likelihood method for reconstructing tree topologies. Molecular Biology and Evolution.

[b90-ebo-03-341] Swofford DL (2002). PAUP* 40: Phylogenetic Analysis Using Parsimony (*and Other Methods), version 4.

[b91-ebo-03-341] Swofford DL, Olsen GJ, Hillis DM, Moritz C (1990). Phylogeny reconstruction. Molecular systematics.

[b92-ebo-03-341] Swofford DL, Olsen GJ, Waddell PJ, Hillis DM, Hillis DM, Moritz C, Mable BK (1996). Phylogenetic inference. Molecular Systematics.

[b93-ebo-03-341] Tehler A, Little DP, Farris JS (2003). The full-length phylogenetic tree from 1551 ribosomal sequences of chitinous fungi, Fungi. Mycological Research.

[b94-ebo-03-341] Varón A Vinh LS Bomash I Wheeler WC 2007 POY 4.0 Beta release 2205. American Museum of Natural HistoryProgram and documentation available at http://research.amnh.org/scicomp/projects/poy.php

[b95-ebo-03-341] Vos RA (2003). Accelerated likelihood surface exploration: the likelihood ratchet. Systematic Biology.

[b96-ebo-03-341] Wagner WH (1961). Problems in the classification of ferns. Recent Advances in Botany.

[b97-ebo-03-341] Wheeler WC, Novacek MJ, Wheeler QD (1992). Extinction, sampling, and molecular phylogenetics. Extinction and phylogeny.

[b98-ebo-03-341] Wheeler WC (1995). Sequence alignment, parameter sensitivity, and the phylogenetic analysis of molecular data. Systematic Biology.

[b99-ebo-03-341] Wheeler WC (2003). Iterative pass optimization of sequence data. Cladistics.

[b100-ebo-03-341] Wheeler WC, Aagesen L, Arango CP, Faivovich J, Grant T, D’Haese C, Janies D, Smith WL, Varón A, Giribet G (2005). Dynamic homology and phylogenetic systematics: a unified approach using POY.

[b101-ebo-03-341] Wheeler WC, Giribet G, Edgecombe GD, Cracraft J, Donoghue MJ (2004). Arthropod systematics. The comparative study of genomic, anatomical, and paleontological information. Assembling the Tree of Life.

[b102-ebo-03-341] Wheeler WC, Gladstein D, De Laet J (2002). POY version 3.0, version Program and documentation available at ftp.amnh.org/pub/molecular. American Museum of Natural History.

[b103-ebo-03-341] Whiting MF, Carpenter JM, Wheeler QD, Wheeler WC (1997). The Strepsiptera problem: phylogeny of the holometabolous insect orders inferred from 18S and 28S ribosomal DNA sequences and morphology. Systematic Biology.

[b104-ebo-03-341] Williams TL, Smith ML (2006). The role of diverse populations in phylogenetic analysis.

[b105-ebo-03-341] Zwickl DJ 2006 Genetic algorithm approaches for the phylogenetic analysis of large biological sequence datasets under the maximum likelihood criterionPhD Thesis, The University of Texas at Austin, Austin115

[b106-ebo-03-341] Zwickl DJ, Hillis DM (2002). Increased taxon sampling greatly reduces phylogenetic error. Systematic Biology.

